# Restoration of the Jaw

**Published:** 1869-06

**Authors:** J. D. Patterson

**Affiliations:** Lawrence, Kansas.


### ARTICLE IV.

### *Restoration of the Jaw.*

By Dr. J. D. Patterson, of Lawrence, Kansas.

The patient, Major J. E. Montandon, of Oskaloosa Kansas, had an operation performed for necrosis of the left superior maxilla, the whole of the maxilla being removed from the right central incisor, also part of the hard palate as the model sent will show.

The operation was performed by the late Dr. Mussey of Cincinnati, Ohio, some eighteen years since, and was extensively noticed at the time by the medical news of the day. After such an operation considerable deformity of course existed, rendering the substitution of an artificial part very desirable; and notwithstanding the fact that many operators had failed, I advised that the operation of substitution was practicable.

Some dentists had advised the severing of the masseter muscle, that being the chief obstacle to a successful operation, against this, however, Dr. Mussey protested and told the patient rather to remain without a plate. I took the impression with plaster, shaping a common cup with wax to suit the case, and, after considerable difficulty, succeeded in obtaining a correct impression of the parts. I removed the plaster as soon as it was hard enough to retain the form, on account of the remaining teeth on the right side permitting the plaster to break, and afterwards united the pieces. I of course made the plate of vulcanite, supplying the artificial jaw with teeth, clasping the only right bicuspid and the central incisor with well fitting gold clasps; the method of procedure I suppose is well known to all practitioners. I also permitted a rubber band around the wisdom tooth, deeming that clasping three teeth instead of one would relieve any strain on the clasped teeth.

I also used a moderate sized air-chamber. The result is in all respects entirely satisfactory, the plate fitting well and firmly restoring the contour of the face, assisting very materially in speech, and as the lower teeth are quite good it improves mastication greatly. Were it not for the drooping of the lip on the left side on account of the attachment of the Superior-alæque-nasi muscle being gone, the face would appear quite natural. I am now satisfied that the plate can after a time be worn without clasps—removing the only objectionable feature.

The patient, who is a gentleman of culture, is highly pleased with the appliance, and finds after a month's trial that not the least inconvenience is experienced from it.

---

